# Applying HFMEA to reduce the incidence of puerperal infection after cesarean section converted from trial of vaginal labor

**DOI:** 10.3389/fmed.2026.1831979

**Published:** 2026-09-07

**Authors:** Yuehong Ren, Yunhe Li, Fang Yuan, Wei Yang, Qingmei Zhang, Jie Zhang

**Affiliations:** 1 Infection Control Office, Baoding Maternal and Child Health Hospital, Baoding, Hebei, China; 2 Infection Control Office, Baoding Hospital of Beijing Children's Hospital Affiliated to Capital Medical University, Baoding, Hebei, China

**Keywords:** cesarean section converted from trial of vaginal labor, HFMEA, infection prevention and control, puerperal infection, surgical site infection

## Abstract

**Objective:**

To explore the efficacy of healthcare-associated infection (HAI) prevention and control management that is based on Healthcare Failure Mode and Effect Analysis (HFMEA) in the prevention of puerperal infection after cesarean section converted from trial of vaginal labor.

**Methods:**

An HFMEA team was set up. The study was conducted from July to December 2023 (pre-intervention) and January to June 2024 (post-intervention), with 115 and 118 puerperae in the pre- and post-intervention periods, respectively. By mapping out the management process of cesarean section converted from trial of vaginal labor, key failure modes related to infection prevention and control were identified. Subsequently, potential causes of these failure modes were analyzed, and improvement plans were formulated, implemented, followed by the evaluation of their efficacy.

**Results:**

Following the optimization of the management process for cesarean section converted from trial of vaginal labor using HFMEA, the Risk Priority Number (RPN) of potential failure modes leading to puerperal infection after this procedure was significantly reduced, with a notable improvement rate. The hand hygiene compliance rate increased from 60.9% (67/110) to 80.4% (90/112), the accuracy rate of surgical hand disinfection rose from 66.7% (80/120) to 90.7% (136/150), and the incidence of puerperal infection after the procedure decreased from 7.8% (9/115) to 1.7% (2/118), with statistically significant differences (*P* = 0.031). In contrast, the surgical site infection rate decreased from 1.7% (2/115) to 0% (0/118), with no statistically significant difference (*P* = 0.155).

**Conclusion:**

The application of HFMEA, as part of a multifaceted quality improvement initiative, was associated with a refinement of the management process for cesarean section converted from trial of vaginal labor and a lower incidence of puerperal infection. However, due to the before-and-after study design, causality cannot be definitively established.

## Introduction

During trial of vaginal labor, in cases where puerperae fail to complete delivery smoothly due to arrest of labor, fetal distress, or other causes, conversion to cesarean section is required, accounting for 10–20% of all puerperae undergoing trial of vaginal labor (1). A 2025 study from Pakistan revealed that the incidence of puerperal infection in the group with failed vaginal birth after cesarean section (VBAC) reached as high as 23.8%, which was significantly higher than that of the successful VBAC group (3.7%) (2). Puerperal infection not only prolongs the duration of hospital stay and increases medical expenses but also may induce severe systemic infection, sepsis, and even death (3). Furthermore, it is closely associated with adverse pregnancy outcomes, including neonatal infection, preterm birth, and low birth weight (4). Therefore, adopting effective measures to prevent puerperal infection after cesarean section converted from trial of vaginal labor is clinically significant.

Healthcare Failure Mode and Effect Analysis (HFMEA) is a systematic, proactive risk management tool aimed at identifying, assessing, and mitigating potential failure modes and their impacts in medical processes. By integrating interprofessional team collaboration with qualitative and quantitative analysis, HFMEA pinpoints high-risk links and puts forward improvement measures to reduce the probability of medical errors (5). In recent years, HFMEA has exhibited significant efficacy in various medical settings, such as medication management, surgical instrument sterilization, and radiotherapy, emerging as a crucial tool for medical risk management (6). Compared to other quality improvement frameworks like PDCA (Plan-Do-Check-Act) or Six Sigma, which are often used for incremental or outcome-focused improvements, HFMEA was selected for its proactive, process-mapping approach. This is particularly well-suited for the complex, multi-step workflow of a trial of labor converted to cesarean section, as it allows for the systematic identification and mitigation of latent failure modes before they manifest as errors. In our hospital, the baseline incidence of puerperal infection after this procedure was 7.8% (9/115) from July to December 2023. By introducing HFMEA to refine the process of cesarean section converted from trial of vaginal labor, the hospital effectively reduced the incidence of puerperal infection.

## Materials and methods

### Establishment of the HFMEA team

This was a prospective, single-center, before-and-after quality improvement study. With the goal of refining the management process of cesarean section converted from trial of vaginal labor, a project team comprising 10 medical and nursing staff from the Infection Control Office, Delivery Room, Operating Room and other departments was established via interdisciplinary collaboration between physicians and nurses. All team members had over 5 years of work experience, among whom 60% held senior professional titles. A Gantt chart was employed to develop the activity plan. The study was conducted at Baoding Maternal and Child Health Hospital, a tertiary teaching hospital. The pre-intervention period was from July to December 2023, and the post-intervention period was from January to June 2024.

### Inclusion and exclusion criteria, and diagnostic criteria

All puerperae who underwent a cesarean section converted from a trial of vaginal labor at the study hospital during the study periods were included. Exclusion criteria were: (1) pre-existing infections at any site before admission; (2) antibiotic use within 48 h prior to admission; and (3) incomplete medical records. Puerperal infection was diagnosed based on the presence of fever (temperature ≥38°C) on two separate occasions within 24 h after the first 24 h postpartum, excluding other causes, accompanied by at least one of the following: uterine tenderness, purulent lochia, or a positive culture from the reproductive tract. Surgical site infection (SSI) was defined according to the CDC/NHSN criteria, involving incisional, deep incisional, or organ/space infections occurring within 30 days post-surgery.

### Process mapping

The team conducted on-site surveys covering multiple links from pregnant women's admission to discharge, and subsequently identified 7 main processes and 21 sub-processes (see Table 1). By reviewing relevant literature (7), the team performed preliminary screening for infection risks among the 7 main processes using the official “HFMEA risk assessment method.” “Antepartum care,” “intrapartum trial of labor” and “cesarean section” were identified as high-risk processes for infection, and these were prioritized for intervention.

**Table 1:** Management process of cesarean section converted from trial of vaginal labor.

Main process	Sub-processes
Obstetric admission and reception	Completion of admission procedures
	Disease assessment
	Completion of various examinations
Antepartum care	Pre-induction risk assessment
	Induction of labor
	Dynamic monitoring
Intrapartum trial of labor	Delivery room reception
	Midwife physical examination
	Delivery preparation
	Trial of labor
Failed trial of labor	Puerpera assessment
	Transfer to operating room
Cesarean section	Operating room reception
	Preoperative preparation
	Cesarean section performance
	Postoperative assessment
Postpartum recovery	Handover of the puerpera and neonate
	Maternal-neonatal placement
	Postoperative treatment and nursing
Discharge	Pre-discharge assessment
	Discharge health education

### Hazard scoring

By adopting the brainstorming method, the team enumerated all potential failure modes in the three sub-processes of “antepartum care,” “intrapartum trial of labor,” and “cesarean section.” A hazard matrix was developed, with both the severity (S) and probability of failure (D) of each failure mode classified into 4 levels and assigned scores from 1 to 4 (8) (see Table 2). Meanwhile, a hazard score (S × D) was allocated to each failure mode, and a score of RPN ≥ 8 indicates a high-risk failure mode.

**Table 2:** Hazard matrix table.

Classification	Severity of impact		
Probability of occurrence	Catastrophic	Major	Moderate
Frequent (occurred several times within 1 year)	16	12	8
Occasional (occurred several times within 1, 2 years)	12	9	6
Uncommon (occurred once within 2–5 years)	8	6	4
Rare (occurred once within 5–30 years)	4	3	2
**Catastrophic**	**Major**	**Moderate**	**Minor**
Severity classification			
Outbreak of puerperal infection involving more than 5 cases or personal injury consequences in 3 or more cases due to the outbreak	Outbreak of puerperal infection	Puerperal infection	Potential hazard event; no puerperal infection

### Decision tree analysis

The team utilized a decision tree analysis to appraise the effectiveness of existing control measures for high-risk failure modes and their detectability following occurrence. For failure modes with an RPN score of < 8, the failure point was first evaluated to ascertain whether it constituted a “single weakness” in the overall process. Subsequently, decisions were made based on control measure effectiveness and detectability, and targeted interventions were ultimately designated for 10 failure modes (9).

### Analysis of causes for failure modes

The research team analyzed the potential causes of these 10 failure modes from the 6M perspectives (Man, Machine, Material, Method, Environment, Measurement). Guided by the decision tree analysis, further interventions were ultimately designated for 18 potential causes (see Table 3).

**Table 3:** Scoring and decision tree analysis of infection-related failure causes in cesarean section converted from trial of vaginal labor.

Failure mode	Cause	Hazard score			Decision tree analysis			Action required
		Severity	Probability of failure	RPN	Single weakness	Effective control	Detectability	
Irregular cervical ripening operation	Junior physicians were unaware of the operational protocol	2	3	6	Y	Y	Y	N
	Unclear steps (e.g., hand hygiene) in the operational protocol	2	4	8	→	N	N	Y
Failure to perform surgical hand disinfection before artificial rupture of membranes	Physicians were unaware that surgical hand disinfection is mandatory for this procedure	2	4	8	→	N	N	Y
Gloves contamination or lack of hand disinfection during vaginal examination	Misconception that gloves eliminate the need for hand disinfection	3	2	6	Y	N	N	Y
	Insufficient hand hygiene awareness	3	3	9	→	N	N	Y
	Hand hygiene steps not separately specified in the process	3	4	12	→	N	N	Y
Inadequate perineal irrigation or disinfection	Unskilled operation by junior nurses	2	4	8	→	N	N	Y
	Lack of diagrams for the “three-step irrigation method”	2	4	8	→	N	N	Y
Excessive frequency of vaginal examinations	Junior physicians unclear about examination criteria	2	4	8	→	Y	Y	S
	Impatient inquiries from family members, prompting repeated examinations for appeasement	2	4	8	→	N	N	Y
	No reminder for examination frequency	2	4	8	→	N	N	Y
Failure to implement “one patient, one use, one disinfection” for fetal heart probes	No disinfectant wipes in the operating room, hindering probe disinfection	3	3	9	→	N	N	Y
Inadequate cleaning and disinfection of operating room environment	Cleaners not receiving specialized operating room training	3	3	9	→	N	N	Y
	Lack of SOP for operating room cleaning and disinfection	3	3	9	→	N	N	Y
Insufficient assessment of maternal labor progress	Hasty admission procedures leaving no time for comprehensive assessment	3	2	6	Y	Y	Y	S
	No explicit requirement for comprehensive assessment in the converted cesarean section process	3	3	9	→	N	N	Y
Irregular surgical hand disinfection by operating table staff	Deep-rooted “green channel” concept leading to omitted hand disinfection	2	3	6	Y	N	N	Y
	Prioritizing convenience in emergency situations	2	3	6	Y	N	N	Y
	Management gaps and lack of quality control	2	3	6	Y	N	N	Y
Failure to strictly implement sterile technique during surgery	Unclear responsibilities of circulating nurses, resulting in inadequate supervision of sterile technique	2	3	6	Y	N	N	Y
	Lack of SOP for intraoperative sterile technique	2	4	8	→	N	N	Y

Action required: Y, Yes, intervention required; N, No intervention required; S, Stop, consider further action based on single weakness. RPN, Risk Priority Number; SOP, Standard Operating Procedure.

### Implementation of improvement actions

Targeting the causes of key failure modes, the team adopted the 5W1H method to develop and roll out improvement actions, and determined the type of action to be implemented. The “elimination” plan was accorded top priority; in cases where the problem could not be eliminated, a “control” plan was adopted to facilitate early detection and easy recognition. When no remedial interventions were available, an “acceptance” plan was selected (10). Following extensive deliberations among team members, homogeneous countermeasures were integrated into five major strategies (see Table 4).

**Table 4:** Improvement action plan for the surgical process of cesarean section converted from trial of vaginal labor.

Countermeasure group	Cause of failure mode	Action type	Countermeasure plan	Responsible department	Implementation site
Countermeasure 1	2B6a unskilled operation by junior nurses 2C1b Repeated examinations by physicians to appease impatient family members 2C1c No reminder of the number of examinations	Elimination	Improve the prenatal nosocomial infection risk assessment system: (1) Assess the number of vaginal examinations received by puerperae and whether intravaginal drug/balloon induction, artificial rupture of membranes, or other operations are performed. Puerperae with >3 vaginal examinations or those who have undergone intravaginal drug/balloon induction, artificial rupture of membranes, etc., are defined as high-risk groups for infection, and vaginal examinations shall be conducted by senior midwives (11) (2) Formulate a pre-delivery examination operation assessment form to record the actual situation of intravaginal examination operations, which is posted at the bedside as a reminder	Maternal and child health department	Delivery room
Countermeasure 2	2B2b Unclear infection control guidelines such as “hand hygiene” in the operating protocol 2B3a Physicians unaware that surgical hand disinfection is mandatory for this procedure 2B4a Misconception that hand disinfection is unnecessary when wearing gloves 2B4d “Hand hygiene” step not separately specified in the process 2B6d Lack of diagrams for the “three-step irrigation method” 3B1b Unclear disinfection sequence in the process 3B2a Touching other items with hands before wearing gloves	Elimination	Revise the diagnosis and treatment process for induction of labor: (1) Add perineal cleaning before operations such as vaginal examination, artificial rupture of membranes, and intravaginal drug/balloon induction: wipe the perineum with 10–20% soap solution gauze at least twice, rinse off the soap solution with warm water, and then perform disinfection (2) Upgrade hand hygiene disinfection for such operations to surgical hand disinfection (3) List all “hand hygiene” steps separately in the process as important reminders, and add the prompt “Reperform hand hygiene and replace gloves if hands or gloves are contaminated”	Maternal and child health department, infection control office	Delivery room
Countermeasure 3	5A4b No explicit requirement for comprehensive assessment in the conversion-to-cesarean section process 5C3a Unclear responsibilities of circulating nurses leading to inadequate supervision of aseptic operations 5C3b Lack of SOPs for intraoperative aseptic technique	Elimination	Improve the cesarean section process: (1) Clarify infection risk assessment requirements for operating room receiving nurses: conduct comprehensive assessment based on the pre-delivery examination operation assessment form from the delivery room, and implement preoperative intervention (vaginal lavage with 0.1% PVP-I disinfectant) for high-risk groups (2) The operating room formulates SOPs for laying sterile instrument tables, transferring surgical instruments and dressings, wearing sterile surgical gowns, and non-touch glove-wearing, followed by full-staff training and assessment (3) Revise circulating nurses' job descriptions, improve job responsibilities, and strengthen supervision and guidance of intraoperative aseptic operations	Operating room, maternal and child health department	Operating room
	5C3d Frequent opening of operating room doors causing instantaneous air pressure difference imbalance	Control			
Countermeasure 4	2B4b Insufficient hand hygiene awareness 3C1bManagement gaps and lack of quality control 5C2a Deep-rooted “green channel” concept leading to omitted hand disinfection	Control	Improve the hand hygiene management strategy: (1) Conduct classified hand hygiene training for staff, and use ATP bioluminescence detectors on-site to test hand hygiene effectiveness (2) Monitor hand hygiene compliance and correctness through unannounced observations of all staff (including anesthesiologists, physicians, midwives, and cleaners) with real-name registration (3) Develop a surgical hand disinfection quality checklist for special supervision and inspection (4) Analyze monitoring results monthly and regularly release them in the department work group	Operating room, infection control office	Operating room
Countermeasure 5	5A1a No disinfection wipes available in the operating room (hindering probe disinfection) 5A3a Cleaners not receiving specialized operating room training 5A3c Lack of SOPs for operating room cleaning and disinfection	Elimination	Improve the cleaning and disinfection management strategy for environmental surfaces and medical equipment: (1) Improve SOPs for consecutive surgery cleaning/disinfection and terminal disinfection in the operating room, and refine the scope to cover all devices and items (2) Operating room cleaning and disinfection shall be led by circulating nurses with cleaner participation, and cleaners can only take up posts after passing pre-job training and assessments (3) Formulate disinfection procedures based on instrument properties, e.g., wipe B-ultrasound and fetal heart rate probes with paper towels to remove coupling agent after use, then disinfect with quaternary ammonium salt disinfection wipes, implementing “one patient, one use, one disinfection” (12)	Operating room, infection control office	Operating room

Codes in the first column (e.g., 2B6a, 2C1b, 5C3d) correspond to the failure modes and their causes listed in Table 3. The action types “Control” and “Elimination” were determined based on the decision tree analysis described in the methods section.

### Statistical methods

The SPSS 27.0 statistical software is utilized for data analysis, with univariate analysis employing the χ^2^ (chi-square) test. A *P*-value < 0.05 was considered statistically significant. No formal sample size or power calculation was performed for this quality improvement project; the sample represents all eligible cases during the respective study periods.

## Results

Following the implementation of HFMEA, the hazard index scores of key failure modes exhibited an overall significant improvement (see Table 5).

**Table 5:** RPN values of key failure modes before and after HFMEA implementation.

Failure mode	Before implementation	After implementation	Improvement rate(%)
Irregular cervical ripening operation	8	4	50.0
Failure to perform surgical hand disinfection before artificial rupture of membranes	8	2	75.0
Gloves contamination or lack of hand disinfection during vaginal examination	6	3	50.0
Inadequate perineal irrigation or disinfection	8	4	50.0
Excessive frequency of vaginal examinations	8	4	50.0
Failure to implement “one patient, one use, one disinfection” for fetal heart probes	9	3	66.7
Inadequate cleaning and disinfection of operating room environment	9	3	66.7
Insufficient assessment of maternal labor progress	6	3	50.0
Irregular surgical hand disinfection by operating table staff	6	2	66.7
Failure to strictly implement sterile technique during surgery	6	4	33.3

Improvement Rate(%), [(Pre-implementation RPN-Post-implementation RPN)/Pre-implementation RPN] × 100%.

Following HFMEA implementation, the hand hygiene compliance rate and surgical hand disinfection accuracy rate exhibited a significant increase compared with pre-implementation levels (*P* < 0.05) (see Table 6).

**Table 6:** Hand hygiene compliance and surgical hand disinfection accuracy rate before and after HFMEA implementation.

Group	Hand hygiene compliance [*n* (%)]	Surgical hand disinfection accuracy rate [*n* (%)]
Before improvement	67/110 (60.9)	80/120 (66.7)
After improvement	90/112 (80.4)	136/150 (90.7)
χ^2^	10.137	24.000
*P*	0.001	< 0.001

Following HFMEA implementation, the incidence of puerperal infection after cesarean section converted from trial of vaginal labor was significantly decreased (*P* < 0.05), whereas no statistically significant difference was observed in the surgical site infection rate (*P* > 0.10) (Table 7). The lack of statistical significance for the reduction in SSI is likely attributable to the low baseline incidence rate (1.7%), which would require a much larger sample size to detect a statistically significant difference (type II error).

**Table 7:** Incidence of puerperal and surgical site infections before and after improvement.

Group	Puerperal infection [*n* (%)]	Surgical site infection [*n* (%)]
Before improvement	9/115 (7.8)	2/115 (1.7)
After improvement	2/118 (1.7)	0/118 (0.0)
χ^2^	4.678	2.018
*P*	0.031	0.155

## Discussion

Puerperal infection is defined as an infection of the reproductive tract and its adjacent tissues following childbirth, typically developing within 6 weeks postpartum (13). Such infections may involve the uterus, surgical incisions, pelvic tissues and other sites, and can progress to systemic sepsis in severe cases (13). Historically, puerperal infection was a leading cause of maternal mortality until the 19th century, when Ignaz Semmelweis markedly reduced the incidence of puerperal fever through the widespread promotion of hand hygiene practices (14). In modern clinical practice, although antibiotic administration has reduced associated mortality, puerperal infection remains a major contributor to maternal morbidity and mortality worldwide (13). Well-recognized high-risk factors for puerperal infection include cesarean section, premature rupture of membranes, prolonged labor and more than five vaginal examinations (15). Perinatal women exhibit alterations in their internal environment and physiological status, which impair the self-purification capacity of the reproductive tract. During childbirth, the reproductive tract establishes direct communication with the external environment, thereby elevating infection risk. Additionally, surgical trauma from cesarean section compromises systemic immune function and disrupts the natural defense mechanisms of the reproductive tract, increasing the likelihood of pathogenic invasion and subsequent reproductive tract infection (16).

A 2021 Dutch study demonstrated that HFMEA combined with computer simulation (HFMEA-CS) was applied to analyze medication adherence in patients with pheochromocytoma throughout the entire hospitalization course from admission to discharge. This integrated approach combines expert consensus with quantitative analysis, markedly enhancing the reliability of complex clinical processes (17). Furthermore, HFMEA identified 64 potential failure modes in the intrahospital transfer of critically ill patients from the emergency department to the intensive care unit (ICU), 17 of which had RPN values >8. Following the implementation of a hierarchical transfer strategy and an intelligent assessment system, the incidence of adverse events was reduced from 19.64 to 7.14% (18). These clinical applications confirm that HFMEA can effectively identify high-risk process steps and develop targeted improvement interventions. A 2024 Italian study indicated that HFMEA detected 23 failure modes in a colorectal cancer screening program; improvements in sample tracking and temperature control reduced the potential risk of errors by 75.9% (19). In 2025, Holmes et al. (20) utilized HFMEA to identify 67 Latent Safety Threats (LSTs), 61.2% of which were common issues across multiple hospitals. These findings underscore the pivotal role of HFMEA in facilitating interdisciplinary collaboration and the standardization of clinical processes. During the study period, there were no changes to the hospital's antibiotic prophylaxis protocols or major labor management guidelines, which strengthens the association between the intervention and the observed outcomes. However, other unmeasured confounders cannot be excluded.

## Conclusion

In this single-center before-and-after study, multidisciplinary HFMEA implementation optimized the perioperative process for cesarean section converted from trial of vaginal labor through systematic risk identification and targeted refinements in infection prevention protocols. Following the intervention, hand hygiene compliance and surgical hand disinfection accuracy improved significantly, accompanied by a marked reduction in puerperal infection rates. However, the lack of a concurrent control group and the relatively small sample size limit causal attribution and generalizability. Future multicenter studies with larger cohorts and controlled designs are warranted to validate these findings. Expanding HFMEA to other obstetric procedures may further enhance the quality and safety of maternal care.

## Data Availability

The raw data supporting the conclusions of this article will be made available by the authors, without undue reservation.
